# Probiotic Effect of *Streptococcus dentisani* on Oral Pathogens: An In Vitro Study

**DOI:** 10.3390/pathogens13050351

**Published:** 2024-04-24

**Authors:** Claudia María Bedoya-Correa, Santiago Betancur-Giraldo, John Franco, Santiago Arango-Santander

**Affiliations:** 1 GIOM Group, Faculty of Dentistry, Universidad Cooperativa de Colombia, Medellin 055421, Colombia; john.francoa@campusucc.edu.co (J.F.); santiago.arango@campusucc.edu.co (S.A.-S.); 2 Faculty of Dentistry, Universidad de Antioquia, Medellin 050010, Colombia; santiago.betancurg@udea.edu.co; 3 Salud y Sostenibilidad Group, School of Microbiology, Universidad de Antioquia, Medellin 050010, Colombia

**Keywords:** probiotic, biofilm, oral pathogens, competition, exclusion, displacement

## Abstract

Probiotics, including *Streptococcus dentisani*, have been proposed as an alternative to re-establish the ecology of the oral cavity and inhibit the formation of pathogenic biofilms. The main objective of this work was to assess the probiotic ability of *S. dentisani* against *Streptococcus mutans*, *Streptococcus mitis*, and *Candida albicans* biofilms. The ability of the strains to form a monospecies biofilm and the probiotic potential of *S. dentisani* using the competition, exclusion, and displacement strategies were determined. All strains were moderate biofilm producers. The ability of *S. dentisani* to compete with and exclude *S. mutans* and *S. mitis* during biofilm formation was not significant. However, *S. dentisani* significantly reduced pathologic streptococcal biofilms using the displacement strategy. Also *S. dentisani* reduced the formation of the *C. albicans* biofilm mainly through competition and displacement. In vitro, *S. dentisani* exhibited probiotic potential to reduce the formation of potentially pathogenic biofilms. Further investigation is required to understand the biofilm-inhibiting mechanisms exhibited by this probiotic strain.

## 1. Introduction

The oral cavity hosts one of the largest and most diverse microbial communities in the human body due to unique environmental conditions that promote microbial adhesion and proliferation [1,2]. It is considered a dynamic ecosystem with environmental fluctuations and multiple interactions where commensal bacteria limit the colonization of pathogens, thus maintaining homeostasis [3,4]. Within the oral cavity, some microorganisms experience a transition from the planktonic to the biofilm state, in which sessile bacteria adhere to surfaces and build colonies. This latter state is generated as planktonic bacteria aggregate and co-aggregate to coordinate the structural formation of the biofilm and facilitate intercellular communication among bacteria through signaling molecules [5,6].

Multispecies communities in the biofilm coexist inside an extracellular polymeric matrix, which acts as a barrier to protect microorganisms from hostile exogenous factors. This is one of the reasons why biofilm cells exhibit distinctive features, such as antibiotic resistance, mechanical stress resistance, pH changes, and higher virulence than planktonic cells [5,7,8,9]. Depending on the composition of the microbiota, variations in the microenvironment, and intrinsic factors in the host, the biofilm might acquire either a profile associated with oral health or a pathogenic profile. Therefore, the composition and function of the biofilm are important characteristics that determine the stability of oral health status or the onset and progression of oral diseases [10].

Currently, the treatment of oral conditions involves unspecific control of the biofilm through mechanical removal or antimicrobial therapy to keep the microbial levels compatible with oral health status. However, these methods have shown poor results due to the multifactorial etiology of such diseases [11,12,13]. Therefore, it is important to implement alternatives to conventional therapeutic and prevention approaches that are oriented at reducing biofilm formation without affecting the ecological balance of the oral cavity. It is in this context that probiotics might play an important role [13,14].

Probiotic species, which are not pathogenic, are defined as living microorganisms that, when administered in the right doses, provide benefits to the health status of the host [15]. It has been demonstrated that probiotics have the potential to modify the environmental conditions and produce changes in the microbiota by competing with other microorganisms for nutrients and specific receptor-binding sites. They also have the ability to inhibit their growth by producing antimicrobial peptides, and some probiotics may participate in the indirect elimination of pathogens by stimulating the host’s immune system through cytokine overexpression, which leads to a higher phagocytic activity. Due to the aforementioned factors, probiotics have been considered an effective option to reduce the incidence of infections produced by pathogenic biofilms [5,13,16,17].

*Streptococcus dentisani* is a Gram-positive, facultative anaerobic coccus that was first isolated in samples from a dental biofilm in healthy patients without caries history [18,19]. This species has been proposed as a probiotic because it is a natural colonizer in the oral cavity, is innocuous, does not produce toxic secondary metabolites, has the capacity to survive the masticatory process, and is sensitive to the gastric pH. It has also been demonstrated that it codifies multiple peptides, known as bacteriocins, whose antimicrobial activity contributes to the growth inhibition of oral pathogens [19,20]. In addition, when *S. dentisani* detects a reduction in the pH level, it has the ability to express genes that activate the arginine metabolic pathway with the subsequent production of ammonia, thus buffering the acid in the biofilm, which will eventually lead to reducing the growth of acidogenic bacteria [18,19,20]. The use of probiotics in oral health is limited, and few studies have focused on the control of pathogenic biofilm formation by using probiotic species isolated from the oral cavity [21,22,23,24]. Therefore, the main objective of this work was to assess, in vitro, the ability of *S. dentisani* to compete with, exclude, and displace *Streptococcus mutans*, *Streptococcus mitis*, and *Candida albicans* cells during biofilm formation.

## 2. Materials and Methods

### 2.1. Saliva Collection and Sterilization

The saliva used in this work was obtained from a previous in vitro experimental work, which was approved by the Ethics Committee from Universidad Cooperativa de Colombia (Act 003/2022). A total of 15 mL of saliva stimulated by the mastication of 1 g of sterile paraffin was collected. Saliva was obtained from a healthy, caries-free, non-smoking individual without periodontal or systemic conditions. The donor received verbal and written information on the objectives and signed an informed consent form before sample collection. In order to eliminate cell debris, the collected saliva was transferred to Eppendorf tubes and centrifuged (Thermo Fisher Scientific, Waltham, MA, USA) at 12.000 RPM for 10 min at 4 °C. The supernatants were transferred to sterile tubes and centrifuged as already mentioned. Then, the soluble fraction was filtrated through a 0.22 µm sterile membrane syringe filter (New LBSSP E002, Jinan, China). A sterility test was performed by seeding 100 µL of the previously filtrated saliva into BHI agar and incubating it in microaerophilic conditions for 48 h. The sterile saliva was frozen at −20 °C until used.

### 2.2. Reference Strains and Growth Conditions

*S. dentisani* CECT 7746 (Colección Española de Cultivos Tipo, Universitat de Valencia, Valencia, Spain), *S. mutans* ATCC 25175 (American Type Culture Collection, Manassas, VA, USA), *S. mitis* NCIMB 13770 (Microbiologics, St. Cloud, MN, USA), and *Candida albicans* ATCC 10231 (American Type Culture Collection, Manassas, VA, USA) reference strains were used. These strains were kept frozen at −20 °C in 20% glycerol (Sigma-Aldrich, Missouri, MO, USA). *S. dentisani*, *S. mutans*, and *S. mitis* were reactivated in MM (BHI) agar (Difco Laboratories, Le Pont de Claix, France), and *C. albicans* was reactivated in Sabouraud Chloramphenicol (Scharlab S.L., Barcelona, Spain) agar. The strains were incubated at 37 °C in microaerophilic conditions for 18 h in a 5% CO_2_ atmosphere.

### 2.3. Macroscopic Characterization of the Reference Strains

Before the competition, exclusion, and displacement tests, each strain was independently seeded in BHI agar and incubated under microaerophilic conditions at 37 °C for 48 h in a CO_2_ atmosphere. After the incubation period, colonies were macroscopically observed, and photographic registration at 8–32× using a stereomicroscope (Stemi DV4, Zeiss Microscopy, Oberkochen, Germany) was performed. Then, a detailed description of the features and morphological characteristics of the colonies from each strain was carried out. 

### 2.4. Preparation of the Microbial Inoculum

After reactivation of the strains, cell suspensions were prepared with the 18 h cultures by transferring 10 mL of BHI broth (Merck Millipore, Burlington, MA, USA) supplemented with 5% sucrose (Thermo Fisher Scientific, Waltham, MA, USA). Continuous measurements with a turbidimeter (Velp Scientifica, Usmate Velate, Italy) were performed to obtain a turbidity of 90 ± 5 NTU (Nephelometric Turbidity Units), which corresponds to a cell concentration of 1.5–2.0 × 10^8^ CFUs/mL (Colony-Forming Units per milliliter).

### 2.5. In Vitro Evaluation of Monospecies Biofilm Formation of S. dentisani, S. mutans, S. mitis, and C. albicans

In vitro evaluation of monospecies biofilm formation was performed by crystal violet plate microtitration. Flat-bottomed 96-well polystyrene microtitration plates (Costar, Corning Inc., Corning, NY, USA) were used following a protocol previously described by Elexson et al. [25] with some modifications. Briefly, 100 µL of sterile saliva was added to each well and it was homogenized in an orbital shaker (Thermo Fisher Scientific, Waltham, MA, USA) for 3 h at 37 °C to allow the adsorption of protein molecules into the well’s walls. After this period, saliva was gently removed by a pipette and allowed to dry at RT inside a vertical flow chamber (BioBase, Qingdao, China). An amount of 200 μL of cell suspension from each strain was added to four different wells (4-fold replicate) and incubated at 37 °C under microaerophilic conditions for 24 h in a 5% CO_2_ atmosphere. Then, supernatants from each well were discarded and 200 µL of BHI broth supplemented with 5% sucrose was added. Plates were incubated under the mentioned conditions for an additional 24 h. BHI broth supplemented with 5% sucrose was used as a negative control. Forty-eight hours after biofilm formation, the supernatants were discarded, and each well was washed twice with 200 µL of 0.9% saline (Corpaul, Medellín, Colombia) to remove planktonic cells. Plates were allowed to rest for 5 min, and 120 µL of formalin (Prodeysa Ltd.a., Medellín, Colombia) was added for 15 min at RT to fix the biofilms. These assays were performed by triplicate with four replicas per strain. 

### 2.6. Quantification of S. dentisani, S. mutans, S. mitis, and C. albicans Biofilm Formation

Biofilm formation was quantified by a crystal violet assay [25] with some modifications. After biofilm fixation, wells were stained with 150 µL of 0.2% crystal violet (Químicos Albor, Medellín, Colombia) for 15 min. Then, each well was washed twice with 0.9% saline (Corpaul, Medellin, Colombia) until a reduction in the coloration was observed. They were allowed to dry at RT, and 150 µL of 95% ethanol (Merck KGaA, Darmstadt, Germany) was gently added to resolubilize the staining solution bonded to the cells. This procedure was performed for the negative controls as well. After a discoloration period of 30 min, samples were transferred to wells from a fresh microtitration plate, and the concentration of the crystal violet pigment was measured in the decoloring solution using a spectrophotometer (Thermo Fisher Scientific, Waltham, MA, USA) at an optical density (OD) of 570 nm. These assays were performed in triplicate with four replicas per strain. 

### 2.7. Assessment of the Biofilm Formation Ability of S. dentisani, S. mutans, S. mitis, and C. albicans

In order to determine whether the microbial strains could produce a biofilm, the OD cutoff value (ODc) was calculated. This term was defined as the negative control average (OD) + 3× the standard deviation of the negative control. The negative control corresponds to the optical density (OD) of the sterile BHI broth supplemented with 5% sucrose. As this culture medium is not transparent, it emits a specific OD when read by a spectrophotometer that must be considered to determine the ODc. Then, classification of the categories reported in Table 1 was performed to determine each strain’s degree of ability to form a biofilm [26].

### 2.8. Assessment of Reduction in Biofilms from S. mutans, S. mitis, and C. albicans

Competition, exclusion, and displacement tests were performed to assess the ability of *S. dentisani* to inhibit the adhesion and biofilm formation of *S. mutans*, *S. mitis*, and *C. albicans.* Assays were performed in flat-bottomed 96-well microtiter plates that were incubated at 37 °C with sterile saliva for 3 h. Two repetitions and three replicas per repetition were carried out. Previously described protocols [27,28,29], with minor modifications, were followed.

#### 2.8.1. Competition Test

To evaluate the ability of *S. dentisani* planktonic cells to competitively inhibit the biofilm formation of the oral pathogens in this experiment, 100 µL of each pathogenic strain was co-cultured independently with 100 µL of the probiotic strain at the same cell concentration. This included 100 µL of *S. mutans* [1.5–2.0 × 10^6^ UFC/mL] + 100 µL of *S. dentisani* [1.5–2.0 × 10^6^ UFC/mL]; 100 µL of *S. mitis* [1.5–2.0 × 10^6^ UFC/mL] + 100 µL of *S. dentisani* [1.5–2.0 × 10^6^ UFC/mL]; and 100 µL of *C. albicans* [1.5–2.0 × 10^6^ UFC/mL] + 100 µL of *S. dentisani* [1.5–2.0 × 10^6^ UFC/mL. Growth controls were included as positive controls (200 µL of the standard inoculum from each pathogenic strain and the probiotic strain (monospecies biofilms)). As negative controls, or sterility controls, 200 µL of BHI broth supplemented with 5% sucrose was used. Plates were incubated at 37 °C in microaerophilic conditions (5% CO_2_) for 48 h to allow biofilm formation. Two repetitions and three replicas per repetition were carried out. Supernatants were discarded and wells were washed twice with 100 µL of 0.9% saline (Corpaul, Medellín, Colombia) to remove non-adherent cells. Then, 150 µL of 0.9% saline was added to each well and sonication was performed with an ultrasonic sonicator (QSonica Q500, Newtown, CT, USA) at 50% power for 30 s to detach the cells that formed a biofilm. Serial microdilutions (10^−1^–10^−4^) were performed and 100 µL of each dilution was inoculated in BHI agar using the spread plate method. Cultures were incubated at 37 °C in microaerophilic conditions (5% CO_2_) for 48 h and a viable-cell count was then performed. 

#### 2.8.2. Exclusion Test

The exclusion ability of *S. dentisani* biofilm cells against planktonic cells of *S. mutans*, *S. mitis*, and *C. albicans* was assessed. An amount of 200 µL of *S. dentisani* (1.5–2.0 × 10^6^ CFUs/mL) was incubated at 37 °C in microaerophilic conditions (5% CO_2_) for 24 h. Supernatants were carefully discarded, and 200 µL of each pathogen, at the same concentration, was added independently to the pre-established *S. dentisani* biofilms. Plates were incubated again for 24 h following the aforementioned conditions. Growth controls were included as positive controls (200 µL of the standard inoculum from each pathogenic strain and the probiotic strain (monospecies biofilms)). As negative controls, or sterility controls, 200 µL of BHI broth supplemented with 5% sucrose was used. After the incubation period, wells were washed twice with 100 µL of 0.9% saline, and the exact same protocol as reported for the competition test was followed. Two repetitions and three replicas per repetition were carried out.

#### 2.8.3. Displacement Test

This assay was performed to determine the ability of *S. dentisani* planktonic cells to displace *S. mutans*, *S. mitis*, and *C. albicans* pre-established biofilm cells. An amount of 200 µL of the oral pathogens (1.5–2.0 × 10^6^ CFUs/mL) was added to each well and incubated at 37 °C in microaerophilic conditions (5% CO_2_) for 24 h. Supernatants were discarded and 200 µL of *S. dentisani*, at the same concentration, was independently added to each pre-established oral pathogen biofilm. Plates were incubated at 37 °C in microaerophilic conditions (5% CO_2_) for 24 h. Growth controls were included as positive controls (200 µL of the standard inoculum from each pathogenic strain and the probiotic strain (monospecies biofilms)). As negative controls, or sterility controls, 200 µL of BHI broth supplemented with 5% sucrose was used. The washing process and the remaining procedures followed the same protocol as the competition test. Two repetitions and three replicas per repetition were carried out.

To assess the ability of *S. dentisani* to inhibit the biofilm formation of pathogenic strains, the biofilm reduction percentage was calculated using the following equation:

$$\mathrm{Biofilm}\;\mathrm{reduction}\;\%=\frac{\left[CFU\;growth\;control-final\;CFU\right]}{CFU\;growth\;control}\;\times \;100$$

Similarly, a logarithmic reduction scale was used to calculate the variations generated in the viable-cell count (CFUs/mL) of the probiotic strain and pathogenic species after performing the competition, exclusion, and displacement tests. The magnitude of the change in CFUs/mL was determined using the following equation

Log reduction = Log_10_ (CFU growth control) − Log_10_ (final CFU)

where CFU growth control corresponds to the Colony-Forming Units obtained from the monospecies biofilms from each strain, and final CFU corresponds to the Colony-Forming Units obtained after performing the competition, exclusion, and displacement tests. 

### 2.9. Statistical Analysis

After calculating the logarithmic reduction and the percentage of biofilm reduction after the competition, exclusion, and displacement tests, a descriptive analysis was performed by estimating the summary measures (central tendency, dispersion, and position).

In order to compare the growth parameters between *S. dentisani* against each pathogenic strain, Student’s *t*-test for independent variables or a Mann–Whitney U test was performed. In all cases, assessments of compliance with the assumption of homogeneity of variances and of normal distribution with the Levene and Shapiro–Wilk statistical tests, respectively, were carried out. Statistical analyses were performed using IBM^®^ SPSS (V.29) software. *p* values < 0.05 were considered statistically significant.

## 3. Results

### 3.1. Biofilm Formation Ability of S. dentisani, S. mutans, S. mitis, and C. albicans

The assays performed to analyze the monospecies biofilm formation abilities in the microtiter polystyrene plates demonstrated that all of the strains formed biofilms. After incubation for 48 h, *S. dentisani*, *S. mutans*, *S. mitis*, and *C. albicans* formed consistent biofilms and were classified as moderate biofilm producers (Table 2).

### 3.2. Biofilm Reduction of S. mutans, S. mitis, and C. albicans

#### 3.2.1. Competition Test

**Figure 1:**
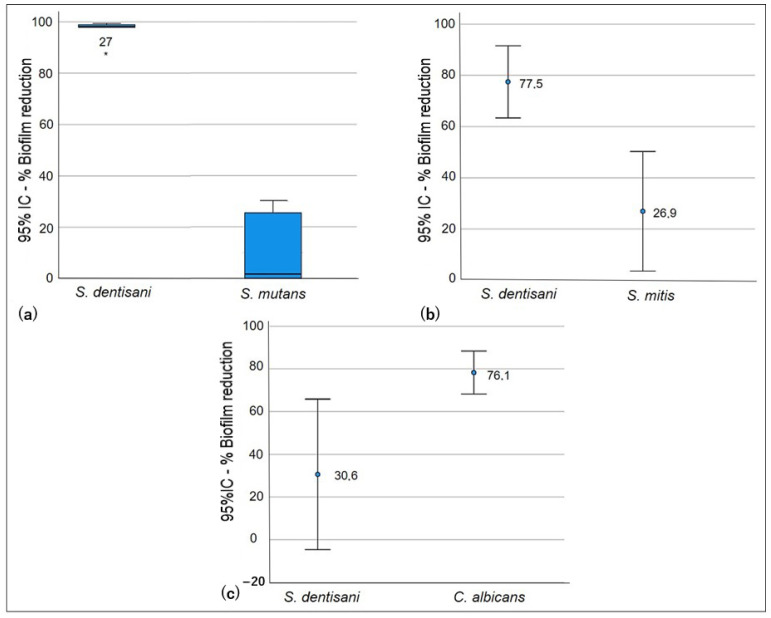
Competition test: ability of *S. dentisani* planktonic cells to reduce biofilms of oral pathogens. *S. dentisani*–*S. mutans* co-culture (**a**), *S. dentisani*–*S. mitis* co-culture (**b**), and *S. dentisani*–*C. albicans* co-culture (**c**).

Significant differences were observed in the competition test (*p* < 0.005) in biofilm formation for all of the evaluated strains (Table 3). The ability of *S. dentisani* planktonic cells to competitively inhibit the formation of *S. mutans* and *S. mitis* biofilms was low. In co-culture, the growth of *S. dentisani* was limited by the streptococcal strains, and higher competition by *S. mutans* was observed, which generated a biofilm reduction percentage of *S. dentisani* that was higher than that of *S. mitis* (98.1% and 77.5%, respectively). However, it was observed that the logarithmic reduction and the percentage of biofilm formation of *S. dentisani* were lower when compared with *C. albicans* since, in this case, *S. dentisani* significantly inhibited *C. albicans* biofilm formation (*p* < 0.05, Figure 1).

#### 3.2.2. Exclusion Test

**Figure 2:**
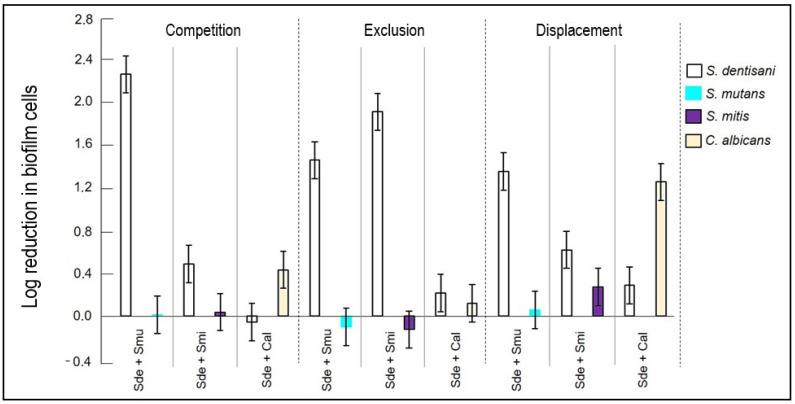
Logarithmic reduction in biofilm cells obtained after the competition, exclusion, and displacement tests. Sde: *S. dentisani*; Smu: *S. mutans*; Smi: *S. mitis*; Cal: *C. albicans*.

**Figure 3:**
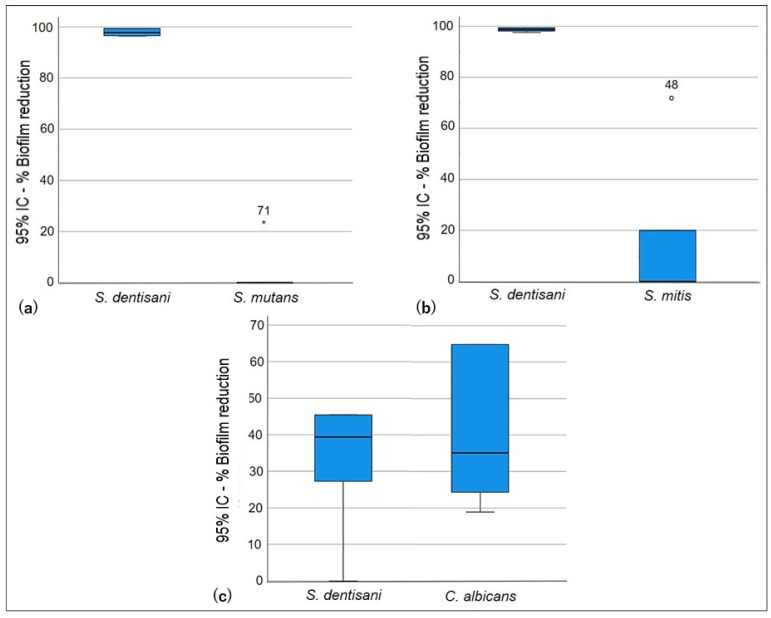
Exclusion test. Exclusion ability of *S. dentisani* biofilm against planktonic cells. (**a**) *S. dentisani*–*S. mutans*, (**b**) *S. dentisani*–*S. mitis*, (**c**) *S. dentisani*–*C. albicans*.

In the exclusion test, significant differences (*p* < 0.005) were only identified when comparing *S. dentisani* biofilm formation against *S. mutans* and *S. mitis* (Table 3). The *S. dentisani* pre-established biofilm did not show the ability to exclude *S. mutans* and *S. mitis* planktonic cells. The latter strains managed to disintegrate the cell cluster of the *S. dentisani* biofilm, causing statistically significant reduction percentages (*p* = 0.005). Figure 2 shows that there was no reduction in the formation of *S. mutans* and *S. mitis* biofilms after the incubation period. Conversely, cell proliferation was observed within these biofilms (Log Red −0.210 and −0.098, respectively) when compared to the growth controls. As for *C. albicans*, the differences in biofilm percentage reduction and logarithmic reduction were not statistically significant (*p* = 0.629). However, it was observed that, due to the exclusion, the production of *C. albicans* biofilm was lower (35.1%) and it could not establish itself as well as *S. mutans* and *S. mitis* (Figure 3).

#### 3.2.3. Displacement Test

**Figure 4:**
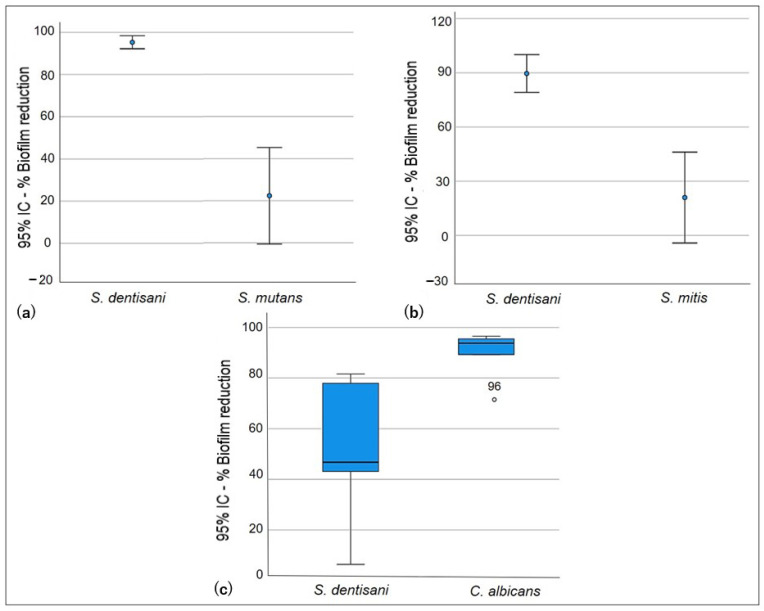
Displacement test. Ability of *S. dentisani* planktonic cells to displace the oral pathogens’ biofilm cells. (**a**) *S. dentisani*–*S. mutans*, (**b**) *S. dentisani*–*S. mitis*, (**c**) *S. dentisani*–*C. albicans*.

In the displacement test, statistically higher inhibition of the formation of the *S. dentisani* biofilm, with respect to *S. mutans* and *S. mitis*, was observed (*p* < 0.005). However, cell proliferation was limited in the presence of *S. dentisani* planktonic cells (Figure 2), although statistically insignificant, and a reduction in the *S. mutans* and *S. mitis* biofilms (22.4% and 21.0%, respectively) was observed in comparison to the exclusion test. On the other hand, the displacement activity of probiotic planktonic cells of *S. dentisani* was effective against *C. albicans* (Figure 4), and a significant reduction in the number of *C. albicans* biofilm cells was observed (*p* = 0.009, Table 3).

## 4. Discussion

Four microbial species that play significant roles within the oral cavity were selected in the current investigation. *S. mutans* and *C. albicans* were selected as pathogen models, *S. mitis* as a primary colonizer, and *S. dentisani* as a probiotic model. *S. mutans* is associated with the onset and progression of dental caries due to its adhesion capacity, biofilm formation abilities, and production of and tolerance to acidic conditions [30]. *C. albicans* is a commensal yeast that may become an opportunistic pathogen. The colonization and persistence of this species within the oral cavity is based on its ability to generate clusters with oral bacteria, especially streptococci, and is related to reduced pH levels, which confirms the ability of *C. albicans* to produce and tolerate acidic conditions [31]. *S. mitis* is a commensal that is well recognized for being one of the main colonizers for biofilm formation and is highly found in the oral cavities of healthy individuals. However, it is considered an accessory pathogen because it provides binding sites that facilitate the colonization and propagation of fungal and bacterial species, thus participating in the formation of multi-species biofilms that increase the virulence of the microbial community [32,33]. Lastly, *S. dentisani* has been proposed as an oral probiotic since it has demonstrated effectiveness in reducing the negative impact of some bacterial species due to its metabolic characteristics [19]. In the current work, after determining a similar ability of all strains to produce and form biofilms, the effect of *S. dentisani* on the biofilm formation of *S. mutans*, *S. mitis*, and *C. albicans* was evaluated in vitro using the competition, exclusion, and displacement strategies.

The competition strategy included the co-culturing of probiotic planktonic cells with pathogen planktonic cells. The action mechanism related to this strategy includes competition for binding sites by using adhesins or specific receptors, or competition for nutrients [5]. This test showed a low ability of *S. dentisani* to inhibit the growth of streptococci species when in co-culture, which demonstrates that both *S. mutans* and *S. mitis* have better competition mechanisms, as was demonstrated in a previous study [34]. The success of the competitive adhesion and biofilm formation of *S. mutans* may be explained by interactions that involve a sucrose-dependent mechanism and a sucrose-independent mechanism. The sucrose-dependent mechanism is associated with glucosyltransferases (GtfB, GtfC, and GtfD), which are enzymes responsible for the synthesis of glucan from sucrose. Glucan’s sticky nature provides bacteria-to-bacteria adhesion and cohesion to the surfaces. In addition, they facilitate adhesion to salivary proteins from the salivary pellicle and resistance to mechanical removal by the host. On the other hand, the sucrose-independent mechanism involves proteins from the cell surface, such as the P1 adhesin anchored to the cell wall (also known as Ag I/II, PAc, SpaP, or antigen B) [35]. The culture medium used in the current investigation was supplemented with 5% sucrose to allow the activation of the sucrose-dependent mechanism for glucan synthesis, thus potentializing the adhesion of *S. mutans* to the salivary pellicle and coaggregation with other bacterial cells. In addition, the cell surface proteins of *S. mutans* allowed it to rapidly adhere to the binding sites of the saliva proteins adsorbed into the wells. The activation of both mechanisms may explain the competition of *S. mutans* as they facilitate the prompt colonization of the surfaces, thus limiting the adhesion of *S. dentisani*.

Adhesion generated by primary colonizers constitutes an initial, critical step in the colonization process. The *S mitis* genome contains several sequences coding different adhesins [36] that have the ability to adhere swiftly and irreversibly to cell receptors in the salivary pellicle on the surface of the tooth and act as anchoring sites for secondary colonizers to bind to [37]. In addition, the ability of *S. mitis* to colonize and maintain multiple bonding sites is due to the expression of a protease that hydrolyzes and inactivates A1 immunoglobulin (IgA1), which facilitates the adherence and initial colonization, toxin synthesis, and modulation of the host’s immune system. These colonization factors allow *S. mitis* to compete for space and nutrients with other microbial communities [38]. 

In a pioneering study aimed at establishing the probiotic action of *S. dentisani*, the ability of this strain to colonize the oral cavity and its antimicrobial potential over oral pathogens were demonstrated. After performing inhibition assays with concentrated supernatants (free of cells) of *S. dentisani* cultures on Gram-positive and Gram-negative bacteria from the oral cavity, it was reported that the simultaneous incubation of such supernatants with bacterial cultures produced a significant inhibitory effect on the growth of *S. mutans* and *Streptococcus sobrinus*. In addition, SEM images showed that *S. dentisani* supernatants produced pores on the cell wall of *S. mutans*, changes in the structure of the *Prevotella intermedia* cell wall, and cell lysis in *Fusobacterium nucleatum* [19]. These results, however, were not confirmed in the current investigation, since the assays were performed using *S. dentisani* cell suspensions instead of supernatants that contain high concentrations of bacteriocins and other cell subproducts that may have a direct effect on the viability of *S. mutans* and *S. mitis*.

The exclusion test consists of pre-coating a surface with probiotic biofilm to inhibit the adhesion of pathogenic planktonic cells by creating a protective barrier that reduces the availability of binding sites, thus blocking the adhesion of pathogens [5]. In the current study, *S. mutans* and *S. mitis* cells exhibited an ability to alter the *S. dentisani* biofilm architecture, causing high percentages of biofilm reduction. In addition to excluding probiotic cells, these strains increased their cell density inside the biofilm.

A variety of mechanisms have evolved in *S. mutans* and *S. mitis* that allow them to successfully colonize hostile environments, such as the oral cavity. One of these mechanisms is known as quorum sensing, which is a gene-regulated method that is cell-density-dependent. When chemical signs from the environment are detected, the synthesis of antimicrobial peptides, cell proliferation, and the response to stress are regulated [39,40,41,42]. In this study, after the implementation of the exclusion strategy, *S. mutans* and *S. mitis* cells established and adapted to the environment to initiate biofilm formation, and when a considerable cell density was achieved, these strains possibly modulated systems to regulate their growth and activated competition genes to synthesize mutacin and other antimicrobial peptides, thus causing *S. dentisani* cells to die and the disruption of the *S. dentisani* biofilm. The response of *S. dentisani* to this hostile environment created by streptococcal species is yet to be understood, and even though the ability of *S. dentisani* to produce bacteriocins of a peptide nature has been previously defined [19,43], the response of this probiotic to biofilm-forming bacterial clusters is not clear.

Even though no significant differences in the percentage of reduction in *C. albicans* biofilm using the exclusion strategy were found, it was possible to observe that *C. albicans* biofilm production was reduced and it could not establish itself as well as *S. mutans* and *S. mitis* biofilms, which is in agreement with previous results [20] where *S. dentisani* did not inhibit the growth of *C. albicans* completely, but it showed an effect on growth speed, thus generating a reduction in *C. albicans* cell density.

The displacement strategy involves contact between probiotic planktonic cells and pathogen cells from the biofilm. The goal is to cause disruption in the architecture of the pathogenic biofilm [5]. In the current investigation, *S. dentisani* planktonic cells caused a significant reduction in the number of *C. albicans* biofilm cells (93.8%), which demonstrated the displacement ability of *S. dentisani* on *C. albicans* cells. This result may suggest a possible antifungal effect of *S. dentisani* over *C. albicans*. The antifungal effect of probiotic cells, especially lactobacillus *spp*., has been widely investigated. Matsubara et al. [44] assessed the inhibitory effects of *Lactobacillus rhamnosus*, *Lactobacillus casei*, and *Lactobacillus acidophilus* planktonic cells on *C. albicans* biofilm cells at various developmental stages. The results from these authors showed that different lactobacillus species inhibited *C. albicans* biofilm formation by suppressing the initial colonization and hypha formation due, possibly, to metabolites excreted by lactobacillus that destabilize the organization and structure of the *C. albicans* biofilm. These authors also determined that the direct contact of probiotic cells with *C. albicans* biofilm cells is fundamental for the antibiofilm effect during the maturation stage [44]. The *S. dentisani* 7746 probiotic strain has a wide number of bacteriocins with potential to oppose and overcome oral pathogens. The inhibitory effect of these antimicrobial peptides has been evaluated against oral and intestinal streptococci and non-streptococcal oral bacteria, including some species associated with periodontitis [43]. However, the antifungal effect of *S. dentisani* on *C. albicans* is poorly understood. Therefore, additional investigations to understand the fungi–bacteria interaction and the mechanisms of action of *S. dentisani* antimicrobial peptides on *C. albicans* are required.

Like *S. mutans*, *S. mitis* is an acidogenic and aciduric species that has the ability to reduce the pH level below 5.5 when grown in culture medium supplemented with glucose [45]. Lopez-Lopez et al. [19] demonstrated that even though *S. dentisani* is not an acidophilic species, it may grow at pH values between 6.0 and 7.5, which indicates that it may tolerate moderately acidic conditions. In addition, it was reported that the growth of *S. dentisani* in culture media with a pH around 6.0 suggests the activation of a buffering metabolic route. However, this buffering is generated in arginine-containing culture media, where pH levels start increasing after incubation for 12 h, hence reaching the initial pH values. In culture media without arginine, pH values decreased considerably [19]. The culture medium used in the present study was not supplemented with arginine, which may explain the high level of *S. dentisani* biofilm reduction obtained in the displacement test, since *S. dentisani*, possibly, did not have the ability to efficiently counteract pH changes in the medium. In this test, *S. dentisani* planktonic cells were added 24 h after the formation of *S. mutans* and *S. mitis* biofilms. During this incubation time, glucose in the culture medium was probably metabolized by the cells inside the biofilm, causing a reduction in pH levels that led to a reduction in the viability of *S. dentisani*. The aforementioned finding is evidence of the low displacement ability of *S. dentisani* over these two strains. However, even though no significant differences were found, *S. dentisani* planktonic cells did cause a reduction in *S. mutans* and *S. mitis* biofilms, thus demonstrating that the displacement strategy of *S. dentisani* is more efficient than the exclusion approach.

## 5. Conclusions

Regarding the limitations of the current study, in addition to being an in vitro approach and the fact that the culture medium was not supplemented with arginine, this study only assessed one strain per species, and the high intra-species genotypic variability was not considered. Therefore, these results may be species-specific. However, this work demonstrated that *S. dentisani* reduced the formation of the *C. albicans* biofilm mainly through competition and displacement. In addition, even though the ability of *S. dentisani* to compete with and exclude *S. mitis* and *S. mutans* during biofilm formation was not significant, it was observed that *S. dentisani* considerably reduced these streptococcal biofilms using the displacement strategy. The results of the current investigation showed the ability of *S. dentisani* to reduce the biofilm formation of potentially pathogenic species. Therefore, it is recommended to continue this line of work to understand, in depth, the probiotic mechanisms of *S. dentisani* to elucidate the role of this species in maintaining the balance among the microbiota of the biofilm and oral health.

## Figures and Tables

**Table 1:** Strain classification according to the ability to form a biofilm.

Result of Calculation	Category
ODcref ≤ ODc	Non-forming
ODc < ODcref ≤ 2 × ODc	Weak
2 × ODc < ODcref ≤ 4 × ODc	Moderate
4 × ODc < ODcref	Strong

ODc: cutoff value; ODcref: optical density of the reference strains.

**Table 2:** Biofilm formation ability of *S. dentisani*, *S. mutans*, *S. mitis*, and *C. albicans*.

Strain	ODcref	Odc Value	Result of Calculation	Category
	(Mean ± SD)			
*S. dentisani*	0.506 ± 0.12	0.179	0.358 < 0.506 ≤ 0.719	Moderate
*S. mutans*	0.552 ± 0.07		0.358 < 0.552 ≤ 0.719	Moderate
*S. mitis*	0.702 ± 0.23		0.358 < 0.702 ≤ 0.719	Moderate
*C. albicans*	0.446 ± 0.02		0.358 < 0.446 ≤ 0.719	Moderate

ODc: cutoff value; ODcref: optical density of the reference strain.

**Table 3:** Biofilm reduction by competition, exclusion, and displacement.

Competition		Log Red	*p*-Value	% Biofilm Reduction	*p*-Value
*S. dentisani*	Median (IQR)	1.730 (1.67–1.97)	0.004 *^a^	98.1 (97.8–98.9)	0.004 *^a^
*S. mutans*		0.005 (−0.29–0.13)		1.6 (0.0–25.5)	
*S. dentisani*	Mean ± SD	0.728 ± 0.314	0.005 *^b^	77.5 ± 13.42	0.001 *^b^
*S. mitis*		0.154 ± 0.137		26.9 ± 22.24	
*S. dentisani*	Mean ± SD	0.170 ± 0.269	0.006 *^b^	30.6 ± 33.57	0.02 *^b^
*C. albicans*		0.645 ± 0.163		76.1 ± 8.84	
**Exclusion**		Log Red	*p*-Value	% Biofilm Reduction	*p*-Value
*S. dentisani*	Median (IQR)	1.660 (1.46–2.22)	0.005 *^a^	97.8 (96.6–99.4)	0.005 *^a^
*S. mutans*		−0.210 (−0.30–−0.10)		0.0 (0.0–0.0)	
*S. dentisani*	Median (IQR)	1.916 (1.74–2.22)	0.006 *^a^	98.8 (98.2–99.4)	0.005 *^a^
*S. mitis*		−0.098 (−0.11–−0.09)		0.0 (0.0–0.0)	
*S. dentisani*	Median (IQR)	0.217 (0.14–0.26)	0.629 ^a^	39.4 (27.3–45.6)	0.629 ^a^
*C. albicans*		0.195 (0.12–0.45)		35.1 (24.3–64.9)	
**Displacement**		Log Red	*p*-Value	% Biofilm Reduction	*p*-Value
*S. dentisani*	Mean ± SD	1.398 ± 0.274	<0.005 *^b^	95.3 ± 2.9	<0.005 *^b^
*S. mutans*		0.126 ± 0.144		22.4 ± 21.8	
*S. dentisani*	Mean ± SD	1.190 ± 0.492	0.002 *^b^	89.6 ± 10.0	<0.005 *^b^
*S. mitis*		0.120 ± 0.154		21.0 ± 24.0	
*S. dentisani*	Median (IQR)	0.275 (0.25–0.66)	0.009 *^a^	46.8 (43.1–78.0)	0.009 *^a^
*C. albicans*		1.210 (0.97–1.35)		93.8 (89.3–95.5)	

* *p* < 0.05. ^a^ Mann–Whitney U test. ^b^ Student’s *t*-test for independent samples. Log Red: logarithmic reduction; IQR: interquartile range; % biofilm reduction: percentage of biofilm reduction.

## Data Availability

The data presented in this study are available from the corresponding author upon request.
